# The attenuation of insulin-like growth factor signaling may be responsible for relative reduction in matrix synthesis in degenerated areas of osteoarthritic cartilage

**DOI:** 10.1186/s12891-021-04096-w

**Published:** 2021-02-27

**Authors:** Nobuho Tanaka, Hirotaka Tsuno, Satoru Ohashi, Mitsuyasu Iwasawa, Hiroshi Furukawa, Tomohiro Kato, Naoshi Fukui

**Affiliations:** 1 Clinical Research Center, National Hospital Organization Sagamihara Hospital, 18-1 Sakuradai, Minami-ku, Kanagawa 252-0315 Sagamihara, Japan; 2 Department of Rheumatology, National Hospital Organization Sagamihara Hospital, 18 − 1 Sakuradai, Minami-ku, Kanagawa 252–0392 Sagamihara City, Japan; 3 Department of Orthopaedic Surgery, National Hospital Organization Sagamihara Hospital, 18-1 Sakuradai, Minami-ku, 252-0392 Sagamihara City, Kanagawa Japan; 4 Department of Rheumatology, National Hospital Organization Tokyo National Hospital, 3-1-1 Takeoka, Kiyose, 204-8585 Tokyo, Japan; 5 Clinical Proteomics and Molecular Medicine, St. Marianna University Graduate School of Medicine, 2-16-1, Sugao, Miyamae-ku, 216-8511 Kawasaki, Kanagawa Japan; 6 Department of Life Sciences, Graduate School of Arts and Sciences, The University of Tokyo, 3-8-1 Komaba, Meguro-ku, 153-8902 Tokyo, Japan

**Keywords:** Osteoarthritis, Chondrocyte, Matrix synthesis, Laser capture microdissection, IGF, IGF1R, IRS1, Sp1

## Abstract

**Background:**

In osteoarthritis (OA), cartilage matrix is lost gradually despite enhanced matrix synthesis by chondrocytes. This paradox may be explained, at least partly, by reduced chondrocyte anabolism in degenerated area of OA cartilage. However, to date, it is not known why chondrocyte anabolism is suppressed in those areas.

**Methods:**

Cartilage was obtained from control knees and end-stage OA knees in macroscopically preserved areas and degenerated areas, and gene expression was analyzed in respective regions of cartilage using laser capture microdissection and qPCR. For the cartilage protein analysis, cartilage was obtained from preserved areas and degenerated areas of OA knees in pairs, and proteins were extracted using urea buffer. Protein concentrations were determined by Luminex and compared between the areas. Cartilage explants prepared from preserved areas and degenerated areas of OA knees were cultured in the presence or absence of an AKT inhibitor, and the gene expression was evaluated by qPCR. Finally, the expression of SP1 was evaluated in OA and control cartilage, and the significance of Sp1 on the expression of IGF1R and IRS1 was investigated in experiments using primary cultured chondrocytes.

**Results:**

Within OA cartilage, the expression of IGF-1, IGF-2, IGF1R and IRS1 was reduced in degenerated areas compared to preserved areas, while the expression of all six IGF-binding protein genes examined was enhanced in the former areas. Consistent results were obtained by a protein analysis. In explant culture, the inhibition of AKT signaling abrogated the abundant matrix gene expression in the preserved areas over the degenerated areas, indicating that suppressed matrix synthesis in degenerated areas may be ascribed, at least partly, to attenuated IGF signaling. Within OA cartilage, the expression of Sp1 was considerably reduced in severely degenerated areas compared to preserved areas, which correlated well with the expression of IGF1R and IRS1. In experiments using primary cultured chondrocytes, the expression of IGF1R and IRS1 was enhanced by the induction of Sp1 expression and reduced by the suppression of Sp1 expression.

**Conclusions:**

The results of this study suggest that attenuated IGF signaling may be responsible, at least partly, for the reduced matrix synthesis in degenerated areas of OA cartilage.

## Background

Osteoarthritis (OA) is the most prevalent joint disease in developed countries and primarily affects the elderly. The loss of cartilage matrix over an extended period of time is a hallmark of the disease. In OA, cartilage matrix is lost gradually by enhanced proteolysis of induced proteinases [1, 2]. However, curiously, anabolic activity of the chondrocytes is highly promoted in OA cartilage [3–6]. Although this contradiction in pathology is often explained by overwhelming catabolism over anabolism, regional differences in chondrocyte metabolism may also explain the paradox of the disease progression. Within OA cartilage, matrix synthesis is enhanced primarily in macroscopically preserved areas, and the enhancement is less obvious, or may not be observed at all, in degenerated areas [3, 6]. In OA, the loss of cartilage matrix occurs primarily at degenerated areas. Therefore, if chondrocyte anabolism is stimulated in degenerated areas, progression of OA can be delayed or prevented entirely. However, at present, there are no clear explanations as to why chondrocyte anabolism is suppressed in degenerated areas of OA cartilage.

In OA, chondrocyte metabolism is regulated by various growth factors and cytokines. Among them, insulin-like growth factor (IGF)-1 is known to have potent anabolic actions on chondrocytes [7–10]. The anabolic potential of IGF-2 has also been shown by recent studies [8, 11]. Previous studies have reported the significance of IGFs in cartilage on health and disease. In normal cartilage, IGF-1 is generated by chondrocytes, and is involved in the maintenance of matrix homeostasis [12, 13]. In OA chondrocytes, the expression of IGF-1 and IGF-2 is enhanced [11, 14, 15] but may not lead to an increase in matrix synthesis because of the reduced response of chondrocytes to these growth factors [16, 17]. Increases in IGF binding proteins (IGFBPs) are considered responsible for this hyporesponsiveness [16–18]. However, despite these previous groups’ efforts, how the IGF system is involved in regional differences in chondrocyte anabolism within OA cartilage remains unclear.

In this study, we explored the mechanisms underlying the regional differences in chondrocyte anabolism within OA cartilage, focusing on IGFs and related molecules. In the analysis, we employed laser capture microdissection (LCM), which is a technology that enables the isolation of a specific area of tissue by its histologic features [19]. Combined with quantitative polymerase chain reaction (qPCR), this technology allowed us to determine the gene expression of respective regions of human cartilage samples in a highly quantitative manner [3, 20]. The results suggest that attenuated IGF signaling may be involved in reduced matrix synthesis in degenerated areas of OA cartilage.

## Methods

### Cartilage samples

This study was conducted in accordance with the Declaration of Helsinki and was approved by the Institutional Review Committee of the National Hospital Organization Sagamihara Hospital. Prior to the acquisition of cartilage samples, informed consent was obtained in writing from each patient or family of the donor. OA cartilage was harvested from a total of 45 end-stage OA knee joints of 45 patients (8 males and 37 females; mean age 74.1 years old; range 63–89 years old) who underwent prosthetic surgery within 4 h after the operation. All knees had medial disease involvement, and the diagnosis of OA was based on the established criteria for knee OA [21].

Control cartilage was obtained from 9 non-arthritic knee joints from 9 donors (6 males and 3 females; mean age 81.1 years old; range 69–88 years old) within 24 h after death. The donors had no known history of joint disease, and the normality of the joint was confirmed macroscopically at the time of harvest.

### LCM and quantitative gene expression analyses

In the analysis, human cartilage tissues were precisely divided into cartilage zones using an LCM device (PixCell IIe; Arcturus, Mountain View, CA, USA), and the gene expression was determined in respective zones. Cartilage obtained from 16 OA knees and 9 control knees was used for this analysis. In OA knees, cartilage samples were harvested from both macroscopically intact areas (preserved areas) and areas showing various degrees of cartilage degeneration (degenerated areas). In control knees, cartilage samples were obtained at 1–3 sites in each knee from the femoral condyles, confirming that the areas showed little sign of cartilage degeneration. At each site, approximately 20 mm × 5 mm of cartilage was obtained in full thickness above the tide mark. The tissue was immediately embedded in OCT compound (Sakura Finetek Japan, Tokyo, Japan), snap-frozen in liquid nitrogen, and stored at -80 °C.

**Fig. 1:**
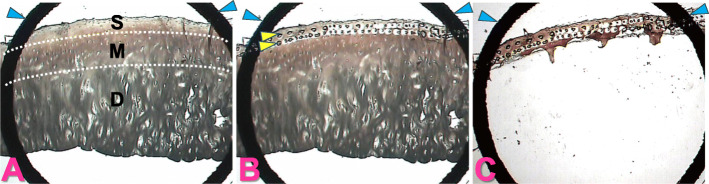
Separation and acquisition of cartilage zone by laser capture microdissection (LCM). **a**, A cryosection prepared for LCM was mounted on a glass slide and set on an LCM device. A transparent plastic film with a transparent plastic base was placed on the section, and cartilage zones were identified through the film and the base based on histological features. S, M and D indicate the superficial, middle and deep zones, respectively. White broken lines indicate borders between cartilage zones. **b**, The zone of interest was fixed to the film by shooting with a laser. The laser was shot multiple times until the entire cartilage zone was attached to the film. The array of spots indicated by yellow arrowheads are the laser shot marks. **c**, After laser shooting, any unnecessary cartilage zones were removed, and only the zone of interest that had become attached to the film was obtained. Acquisition of a superficial zone from a cryosection prepared from preserved areas of OA cartilage is shown. The bold black arcs indicated by blue arrowheads are the mark on the plastic film (Original magnification, X 2)

For analyses, cryosections were prepared from the OCT-embedded cartilage tissues, and cartilage zones were separated by LCM as described previously [3, 20]. In detail, the cryosections were cut into 20- to 40-mm-thick slivers, and first treated with 0.5 M EDTA (pH 8.0) for 3 minutes, before being dehydrated with graded concentrations of ethanol, and clarified with xylene. All reagents were RNase-free, and the entire process was completed within 30 minutes to minimize RNA degradation. The sections were then placed on glass slides, set on an LCM device, and divided into cartilage zones by LCM based on their histological features　[22] (Fig. 1). Immediately after LCM, RNA was extracted from the respective cartilage zones using an RNeasy Micro kit (Qiagen GmbH, Hiden, Germany) with the routine use of DNase I (Qiagen). cDNA was synthesized using Sensiscript reverse transcriptase (Qiagen). The gene expression was evaluated quantitatively by qPCR on a LightCycler (Roche Diagnostics, Basel, Switzerland), using gene-specific primers and probes. SYBR® Premix Ex Taq® Perfect Real Time (Takara Bio, Shiga, Japan) or Premix Ex Taq® Perfect Real Time (Takara Bio) was used for PCR. The cDNA levels were normalized by the expression of *GAPDH*.

### Cartilage protein analyses

Cartilage tissues from 20 end-stage OA knees were used for this analysis. In each knee, cartilage tissues were obtained in pairs from preserved areas and degenerated areas on the tibial plateau. At each area, 400–700 mg wet-weight of tissue was harvested above the tide mark with a scalpel, which was rinsed thoroughly in ice-cold phosphate-buffered saline (PBS). Protein extraction was performed following a previously described method with some modifications [18, 23]. In brief, after being blotted dry and weighed, each cartilage tissue was finely diced and subjected to extraction in 10 ml urea buffer which consisted of 50 mM Tris-HCl buffer, pH 6.0, containing 8 M urea, 0.3 M NaCl, 0.05 % Triton-X100, and proteinase inhibitors (1 mM AEBSF, 0.8 µΜ aprotinin, 40 µΜ bestatin, 14 µΜ E-64, 20 µΜ leupeptin, 15 µΜ pepstatin A). Extraction was carried out at 4 °C for 48 h on an orbital shaker at 100 rpm. The urea buffer was then recovered and clarified by centrifugation, and the supernatant was dialyzed against PBS, pH 7.4, containing the proteinase inhibitors at 4 °C for 72 h with daily change of the buffer. After dialysis, the supernatant was clarified by centrifugation and filtered through a 0.22-mm-pore polyethersulfone filter (Millipore Express; Millipore, Burlington, MA, USA). The filtrate was aliquoted and stored at -80 °C until the analysis.

Protein concentrations in the supernatant were determined by a BioPlex 200 system (BioRad, Hercules, CA, USA) using commercially available kits (Milliplex MAP Total Akt/mTOR Magnetic Bead Kit, and Human IGF Binding protein Magnetic Bead Panel, Millipore). For the measurement, 25 µl of the dialyzed extracts was incubated with a suspension of capture antibody-conjugated magnetic microspheres following the manufacturer’s protocols. The microspheres were then incubated with biotinylated detection antibody and R-phycoerythrin (PE)-conjugated streptavidin, and the fluorescence intensity was measured. Protein concentrations were compared after normalization by the total protein concentration of the extract, which was determined by a Pierce BCA Protein Assay Kit (Thermo Fisher Scientific, Waltham, MA, USA) using bovine serum albumin as a standard.

### Cartilage explant culture

For explant culture, cartilage tissues were obtained from 5 OA knees in pairs from preserved areas and degenerated areas on the tibial plateaus, as described for the cartilage protein analysis. After being rinsed in sterilized PBS, each piece of cartilage tissue was diced into 30–50 cubes of 2–3 mm. The diced cartilage, or explants, were equally divided into two, which were placed onto respective wells of a 12-well plastic plate. These explants were cultured in Dulbecco’s modified Eagle’s medium (DMEM)/F-12 supplemented with ITS Liquid Media Supplement (Sigma Aldrich, St. Louis, MO, USA) and 25 µg/ml ascorbic acid overnight, and then the media was replaced with those containing Akt inhibitor IV (Sigma Aldrich; 5 µΜ in dimethyl sulfoxide [DMSO]) or DMSO alone (vehicle control). After 48 h of culture, the explants were recovered, embedded in OCT compound, snap frozen in liquid nitrogen and stored at -80 °C until analysis. Extraction of RNA from the explants was performed following a previously described method [3]. In brief, 20 µµ-thick cryosections were prepared from the explants embedded in OCT compound, which were immediately immersed in TRIzol (Thermo Fisher Scientific). RNA was first recovered from the TRIzol reagent in the aqueous phase, and then purified using the RNeasy Micro kit (Qiagen).

### Primary culture human articular chondrocytes

To obtain primary culture human chondrocytes, cartilage tissue was obtained from macroscopically intact areas of four OA knee joints, which were subjected to serial enzymatic digestion using Pronase (Sigma Aldrich) and Collagenase P (Sigma Aldrich) [24, 25]. Following digestion, chondrocytes were plated onto a 12-well plastic culture plate at a density of 2 × 10^5^/cm^2^, and maintained in the culture media described above for the explant culture. Chondrocytes were used for experiments starting two days after plating.

### Generation of recombinant adenoviruses

Recombinant adenoviruses were constructed using a ViraPower Adenoviral Expression System (Thermo Fisher Scientific). For this, human *SP1* complementary DNA (cDNA) were cloned into the adenoviral-generating constructs [25]. The construct was then transfected into 293A cells (Thermo Fisher Scientific) using FuGENE 6 (Roche Diagnostics), and the cells were subcultured to generate recombinant adenoviruses carrying these genes under the control of the human cytomegalovirus immediate‐early enhancer/promoter. The viruses were titrated by limiting dilution plaque titration on 293A cells, and used at 50–100 plaque‐forming units/cell. Our preliminary experiments showed that the efficiency of transduction by this method is almost 100 %.

### RNA interference

All small interfering RNAs (siRNAs) were purchased from Qiagen. The siRNAs were introduced into primary cultured chondrocytes by electroporation using a Nucleofector (Lonza, Basel, Switzerland), according to the manufacturer’s protocols with some modifications [25]. In brief, immediately after enzymatic digestion of cartilage tissues, 1 × 10^6^ chondrocytes were suspended in 100 µl of electroporation buffer containing 20 pmoles of siRNA, and the siRNAs were introduced into the cells by electroporation. Chondrocytes were then plated and cultured in DMEM/F-12 containing 20 % fetal bovine serum (FBS), which was replaced with DMEM/F‐12 containing 10 % FBS the next day. Based on the result of our preliminary experiments, the effect of RNAi was evaluated three days after electroporation. In this study, 2 siRNAs with respective sequences were used to suppress the Sp1 expression, which were confirmed to reduce the expression by 43 % and 56 %, respectively, as evaluated by qPCR.

### Statistical analyses

Data were compared by paired or unpaired *t*-tests. For multiple comparisons, data were compared using a one‐way factorial analysis of variance (ANOVA), and when necessary, Fisher’s protected least significant difference was used as a post hoc test. The correlation of the expression was evaluated by a linear regression analysis. *P* values less than 0.05 were considered significant.

## Results

### The expression of IGFs and IGF-related genes is reduced in degenerated areas of OA cartilage relative to preserved areas

**Fig. 2:**
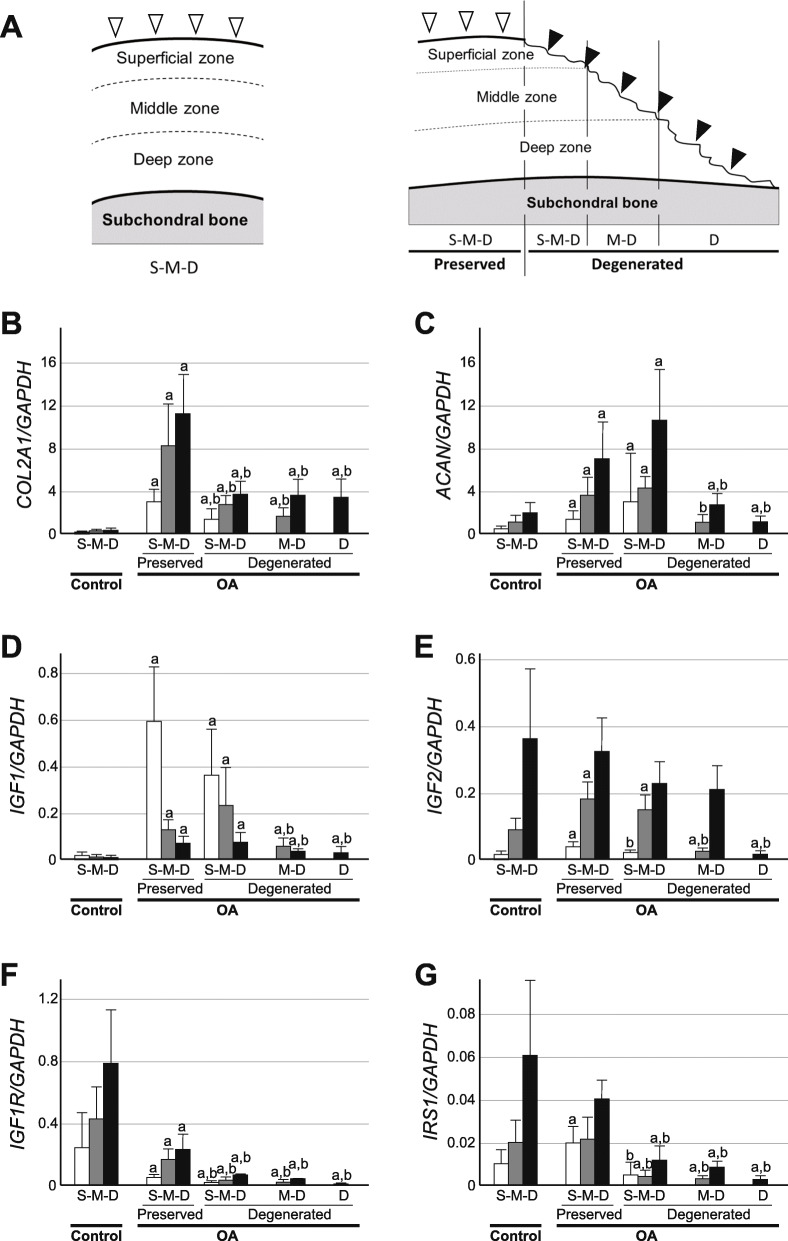
The expression of cartilage matrix genes, IGFs and related genes in OA and control cartilage. Cartilage tissues were obtained from control (Control) and OA knees (OA), and the gene expression was determined in superficial (S), middle (M) and deep cartilage zones (D). **a**, Schematic illustrations of control (left) and OA cartilage (right). While control cartilage retained all three cartilage zones, OA cartilage might retain three (S-M-D), two (M-D) or only one zone (D), as they were obtained from both macroscopically preserved areas (Preserved) and degenerated areas with various degrees of degeneration (Degenerated). Open and closed arrowheads indicate surface of preserved and degenerated cartilage, respectively. **b-g**, The expression of *COL2A1* (**b**), *ACAN* (**c**), *IGF1* (**d**), *IGF2* (**e**), *IGF1R* (**f**) and *IRS1* (**g**) is shown by ratios against the *GAPDH* expression. The findings from superficial, middle and deep cartilage zones are shown by open, shaded and closed bars, respectively. Values are the mean and SE of 7–19 samples. a and b indicate *p* < 0.05 compared to the corresponding zone in control cartilage, or compared to the corresponding zone in preserved areas of OA cartilage, respectively

IGF-1 and IGF-2 require IGF1R and IRS1 to initiate IGF signaling. Therefore, we first investigated the expression of these four genes in OA and control cartilage together with type II procollagen and aggrecan, focusing on regional differences within cartilage. For this analysis, OA cartilage was obtained from macroscopically preserved areas and degenerated areas with various degrees of degeneration, and the gene expression was determined in respective cartilage zones using LCM coupled with qPCR (Fig. 2a).

Compared to the control cartilage, the expression of type II procollagen and aggrecan was highly enhanced in the preserved areas of OA cartilage. Although some discrepancy was noted in the slightly degenerated areas where the three cartilage zones had been retained, the expression of these genes was markedly reduced in the severely degenerated areas where the superficial cartilage zone had been lost due to the disease, compared to the preserved areas (Fig. 2b and c). These results were generally consistent with previous observations [6, 20]. The expression of IGF-1 showed a similar trend, being highly enhanced in the preserved areas of OA cartilage compared to the control but tended to decrease in degenerated areas, particularly in severely degenerated areas (Fig. 2d). In contrast, the expression of IGF-2 did not change much in the preserved areas of OA cartilage from that in the control cartilage, but its expression significantly decreased in the degenerated areas of OA cartilage in the zones exposed to the joint cavity (Fig. 2e).

It is noteworthy that the expression of IGF1R was markedly suppressed in all areas of OA cartilage (Fig. 1f). Within OA cartilage, the IGF1R expression was more markedly reduced in degenerated areas than in preserved areas. The expression of IRS1 did not change much in preserved areas of OA cartilage from that in the control cartilage, but was strongly suppressed in degenerated areas (Fig. 2g). Thus, for all six genes examined here, it was a common trend that the expression was reduced in degenerated areas of OA cartilage compared to preserved areas.

### The expression of IGFBPs is enhanced in degenerated areas of OA cartilage relative to preserved areas

**Fig. 3:**
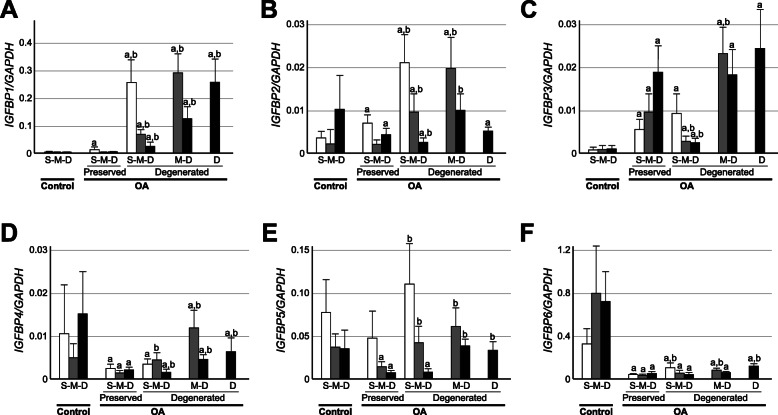
The expression of IGF-BPs in OA and control cartilage. The expression of six IGFBP genes was determined in the respective cartilage zones of control (Control) and osteoarthritic cartilage (OA). The *IGFBP1* (**a**), *IGFBP2* (**b**), *IGFBP3* (**c**), *IGFBP4* (**d**), *IGFBP5* (**e**) and *IGFBP6* (**f**) expression is shown as described in Fig. 1. Preserved and Degenerated indicate macroscopically intact and degenerated areas of OA cartilage, respectively. S, M and D denote superficial, middle and deep cartilage zones, respectively, which are shown by open, shaded and closed bars, respectively. Values are the mean and SE of 7–19 samples. a and b indicate *p* < 0.05 compared to the corresponding zone in control cartilage, or compared to the corresponding zone in preserved areas of OA cartilage, respectively

Next, we investigated the expression of six IGFBP genes in OA and control cartilage in a layer-to-layer manner. In preserved areas of OA cartilage, the expression of IGFBP-1 was significantly elevated only in the superficial zone and not in the middle or deep zone compared to the corresponding zone of control cartilage (Fig. 3a). On the other hand, in the degenerated areas, the expression of IGFBP-1 was dramatically elevated in all areas compared to the control cartilage and preserved areas of OA cartilage. The expression of IGFBP-2 showed a complex pattern of change in OA cartilage. In preserved areas, the expression of IGFBP-2 was significantly elevated in the superficial zone, not significantly changed in the middle zone, and significantly reduced in the deep zone, compared to the corresponding zone of control cartilage (Fig. 3b). In degenerated areas, the expression of IGFBP-2 was significantly enhanced in the superficial and middle zones compared to the control cartilage or preserved areas of OA cartilage, but its expression in the deep zone did not show a consistent trend, being either significantly enhanced or reduced depending on the areas. The expression of IGFBP-3 was significantly enhanced in all areas of OA cartilage, and the enhancement was most obvious in severely degenerated areas where the superficial zone had been lost to the disease (Fig. 3c). For the other three IGFBPs examined here, namely, IGFBP-4, IGFBP-5, and IGFBP-6, the expression decreased in the preserved areas of OA cartilage relative to that in the control cartilage (Fig. 3d-f). However, when compared within OA cartilage, their expression tended to be higher in degenerated areas than in preserved areas. Thus, it was a common trend for all six IGFBP genes that the expression was more elevated in degenerated areas of OA cartilage compared to that in preserved areas.

### IGF1R and IRS1 proteins decrease while IGFBP proteins increase in degenerated areas of OA cartilage relative to preserved areas

Since the gene expression analysis revealed that the expression of IGFs and related molecules differed considerably between preserved areas and degenerated areas within OA cartilage, we next investigated whether or not their protein concentrations differed between those areas. For this analysis, cartilage tissues were obtained from preserved areas and degenerated areas of OA knees in pairs, and respective protein extracts were prepared.

**Fig. 4:**
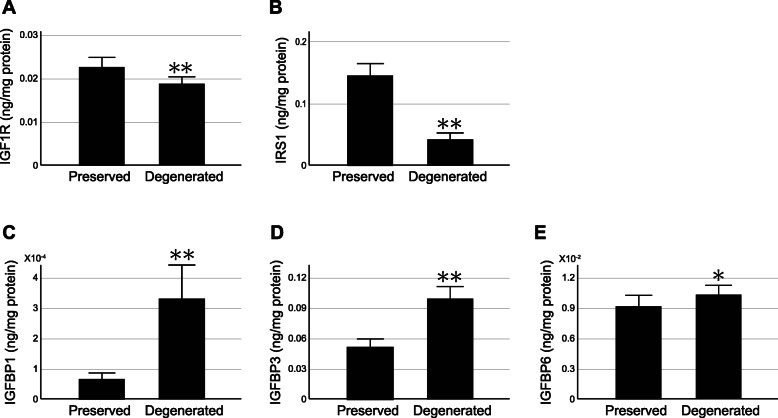
Concentration of IGF-related proteins in OA cartilage. Cartilage was obtained from OA knees at macroscopically preserved areas (Preserved) and degenerated areas (Degenerated) in pairs. Proteins were extracted, and concentrations of indicated protein were determined and compared between the preserved and degenerated areas. The findings for of IGF1R (**a**), IRS1 (**b**), IGFBP-1 (**c**), IGFBP-3 (**d**) and IGFBP-6 (**e**) are shown. Values are normalized by the total protein concentration of the extract. Results are the mean + SE of 20 samples. *, *P* < 0.05 and **, *P* < 0.01

This analysis confirmed that the concentration of IGF1R and IRS1 was indeed reduced in degenerated areas of OA cartilage compared to preserved areas (Fig. 4a and b). This analysis also revealed that the concentrations of IGFBP-1, IGFBP-3 and IGFBP-6 were significantly greater in degenerated areas than in preserved areas, although the difference was less obvious with IGFBP-6 (Fig. 4c-e). The concentrations of the other three IGFBPs were entirely or largely below the detectable levels under our experimental conditions (data not shown).

### Attenuation of IGF signaling could be responsible for the decline in cartilage matrix gene expression in degenerated areas of OA cartilage

Based on the above results, it seemed highly likely that the IGF signaling was attenuated in degenerated areas of OA cartilage. We suspected that this decline in IGF signaling might account for the reduction in the cartilage matrix gene expression in degenerated areas, and conducted experiments to assess that possibility.

In chondrocytes, anabolic activities of IGFs are mediated primarily by AKT signaling [26, 27]. We therefore attempted to estimate the contribution of IGF-1 signaling to chondrocyte anabolism using a specific inhibitor for AKT signaling. For this experiment, cartilage tissues were obtained from preserved areas and degenerated areas of OA knees in pairs. The cartilage was maintained by explant culture in the presence or absence of an AKT inhibitor, and the change in the expression of type II procollagen and aggrecan by the inhibitor was evaluated. Since our preliminary experiments confirmed that the expression of these matrix genes did not change significantly by explant culture for at least five days under our experimental conditions (data not shown), the chondrocytes in the explants were considered to maintain their *in vivo* metabolism during this experiment, where the explants were cultured for 3 days. Thus, the result of this experiment was considered to indicate that the expression of these cartilage matrix genes is sustained by IGF signaling *in vivo* in preserved areas of OA cartilage.

**Fig. 5:**
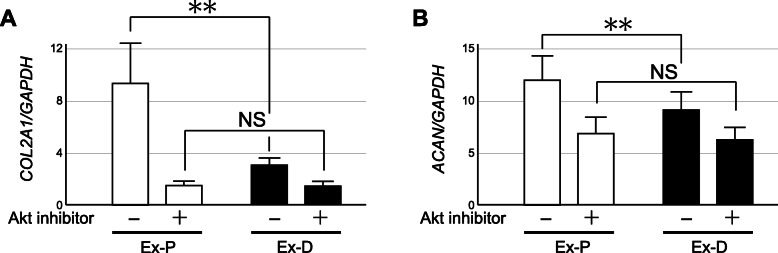
Effects of an AKT inhibitor on the expression of cartilage matrix genes in cartilage explants. Cartilage explants prepared from preserved areas (Ex-P) and degenerated areas of OA cartilage (Ex-D) were cultured under the presence or absence of a specific inhibitor for AKT signaling. Forty-eight hours later, explants were recovered and the expression of type II procollagen (**a**) and aggrecan (**b**) in the explants was evaluated by qPCR. Findings for Ex-P and Ex-D are shown by open and closed bars, respectively. Values are the mean + SE of 5 independent experiments, each duplicate. **, *p* < 0.01. NS denotes not significant

In this experiment, the expression of type II procollagen and aggrecan was maintained at significantly higher levels in the explants from preserved areas (Ex-P) than in those from degenerated areas (Ex-D) after three days of culture (Fig. 5). The AKT inhibitor suppressed the expression of those cartilage matrix genes in both Ex-P and Ex-D. However, the expression suppression by the inhibitor was more obvious with Ex-P, and, as a consequence, the difference in the expression of both cartilage matrix genes became statistically insignificant between Ex-P and Ex-D following inhibitor treatment. This result indicates that IGF signaling is indeed involved in chondrocyte anabolism in OA cartilage, and that the signaling may play a greater role in anabolism in preserved areas than that in degenerated areas. The result in turn implies that the decline in the cartilage matrix gene expression in degenerated areas of OA cartilage can be ascribed, at least in part, to attenuated IGF signaling in the areas. Since the suppressive effect of the AKT inhibitor was more obvious on the type II procollagen expression than on aggrecan in the explants prepared from preserved areas, the type II procollagen expression may be more dependent on IGF signaling than aggrecan in those areas.

### Possible involvement of Sp1 in the regulation of IGF1R and IRS1 expression in OA cartilage

**Fig. 6:**
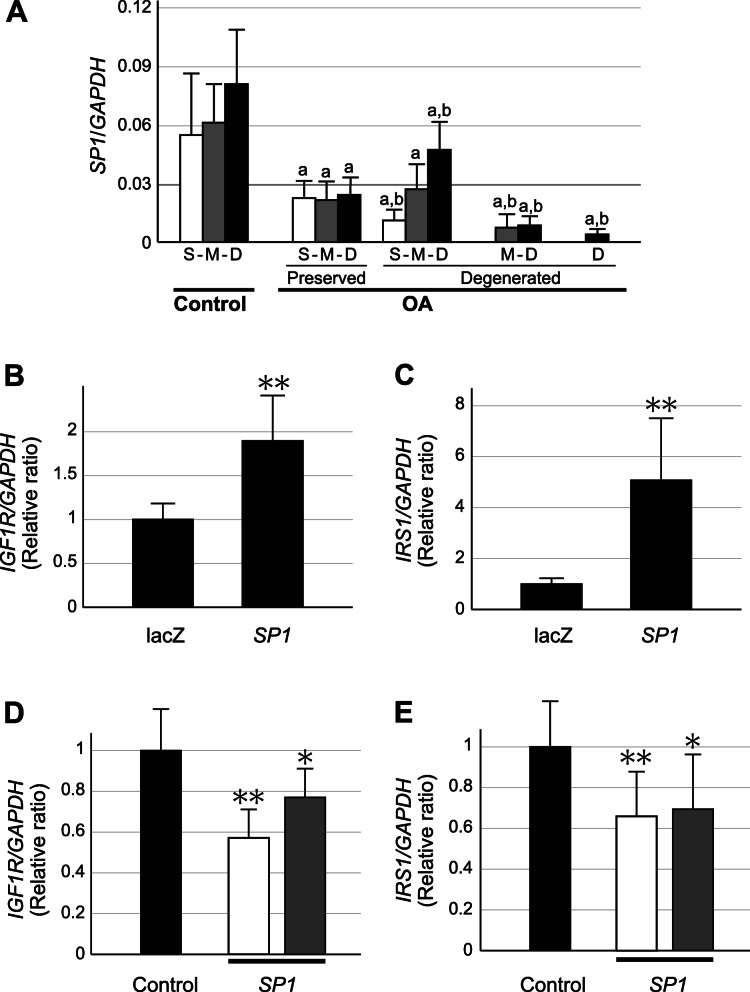
The expression of Sp1 in OA and control cartilage and its significance in the expression of IGF1R and IRS1. **a**, The expression of *SP1* was determined in control (Control) and OA cartilage (OA) in cartilage zones. OA cartilage was obtained from macroscopically intact areas (Preserved) and degenerated areas with various degrees of cartilage degeneration (Degenerated). Results are shown as described in Fig. 1. S, M and D denote superficial, middle and deep cartilage zones, respectively, which are shown by open, shaded and closed bars, respectively. Values are the mean and SE of 6–12 samples. a and b indicate *p* < 0.05 compared to the corresponding zone in control cartilage, or compared to the corresponding zone in preserved areas of OA cartilage, respectively. **b** and **c**, Primary cultured human chondrocytes were infected with adenoviruses encoding *SP1*, and the expression of *IGF1R* (**b**) and *IRS1* (**c**) was evaluated three days later. The expression was normalized to the *GAPDH* expression and compared to the control cells infected with adenoviruses encoding lacZ. **d** and **e**, Two siRNAs for *SP1* were introduced into primary cultured human chondrocytes, and expression of *IGF1R* (**d**) and *IRS1* (**e**) was evaluated three days later. The expression was normalized to the *GAPDH* expression and compared to the control cells given control siRNA. Open and shaded bars represent the results of the two different siRNAs for SP1. In B-E, the results are presented as relative ratios. Values are the mean + SE of 4 independent experiments, each triplicate. **, *p* < 0.01

We next wished to clarify the mechanism underlying the decline in the expression of IGF1R and IRS1 in degenerated areas of OA cartilage. Based on the results of previous studies [28, 29], we suspected that the transcriptional factor Sp1 might be involved in the regulation of the IGF1R expression in OA cartilage, and explored this possibility. We first investigated the expression of Sp1 in OA and control cartilage, and found it to be reduced in the entire area of the OA cartilage but especially in the degenerated areas (Fig. 6a), resembling that of IGF1R (Fig. 2c). We then investigated whether or not Sp1 regulates the expression of IGF-1 in human articular chondrocytes. When the expression of Sp1 was promoted in primary cultured chondrocytes via adenoviral gene transduction, the expression of IGF1R was markedly increased (Fig. 6b). Conversely, the expression of IGF1R was significantly reduced when the expression of Sp1 was suppressed by RNA interference (Fig. 6c). Considering the similarity in the expression pattern within OA cartilage, we evaluated the expression of IRS1 in this experiment, and found that it was also increased or reduced by the induction or suppression of Sp1 expression, respectively (Fig. 6d and e). These results indicate that both the IGF1R and IRS1 expression is regulated by Sp1 in human articular chondrocytes.

### The expression of Sp1 correlates with the expression of IGF1R and IRS1 within OA cartilage

Finally, we investigated the relationship in gene expression among IGF1R, IRS1 and Sp1 within OA cartilage. Previous studies showed that the gene expression may differ considerably among cartilage zones [3, 4]. Therefore, in this analysis, the correlation of the expression was investigated in respective cartilage zones in order to eliminate the bias of zonal difference in the expression.

**Fig. 7:**
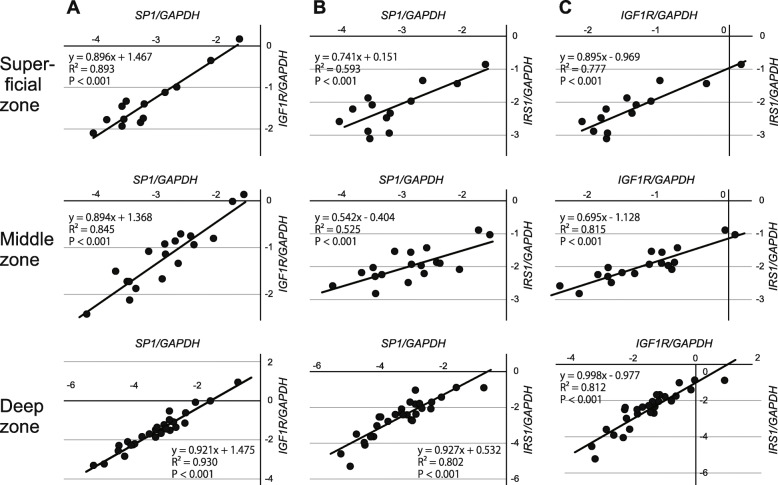
The correlation of the gene expression among IGF1R, IRS1 and SP1. OA cartilage was obtained from preserved and degenerated areas with various degree of cartilage degeneration, and the expression of *IGF1R*, *IRS1*, and *SP1* was determined in cartilage zones by qPCR. The correlation of the expression was evaluated among the three genes in each zone. The relationships of the expression between *IGF1R* and *SP1* (**a**), *IRS1* and *SP1* (**b**), and *IGF1R* and *IRS1* (**c**) are shown on scattergrams together with regression lines, coefficients of determination, and *P* values of correlations. The findings of 29 OA cartilage samples are shown. Upper, middle and bottom panels present results of superficial, middle and deep cartilage zones, respectively. Values are common log transformed

To our surprise, strong positive correlations were observed in all pairs of the three genes, in all three cartilage zones (Fig. 7). This result strengthened the possibility that the expression of IGF1R and IRS1 is regulated together by Sp1 within OA cartilage. Furthermore, the suppression of the IGF1R and IRS1 expression in degenerated areas may have been caused, at least partly, by a reduced Sp1 expression in the areas.

## Discussion

Although an increasing number of studies have been published concerning the pathology of OA, few have focused on the mechanisms underlying the regional difference in chondrocyte metabolism under OA conditions. In this study, we explored this mechanism and obtained data suggesting that a change in the IGF signaling might be involved in these regional differences. Since most of our results were obtained through an analysis of human cartilage samples, they would directly reflect the changes that actually occur in OA patients’ knees. We believe that our findings may provide a new basis for understanding the cartilage pathology in OA.

Several investigators have evaluated the expression of IGF-1 and IGF-2 in OA and control cartilage. An elevated IGF-1 expression in OA cartilage was reported repeatedly by previous studies [15, 30, 31]. Our current analysis confirmed this and further revealed regional differences in the expression, with the expression highly enhanced in preserved areas but modest in moderately and severely degenerated areas. Regarding IGF-2, a previous study reported a reduced expression in OA cartilage [11]. Our current study revealed that the reduction in IGF-2 expression was limited to severely degenerated areas and that IGF-2 was expressed at levels comparable to those in the control cartilage in other areas of OA cartilage.

The expression of IGF1R in OA cartilage has been examined by several investigators, but the results are somewhat conflicting. While one study reported that the expression in OA cartilage was similar to that in control cartilage [32], others reported an enhanced IGF1R expression in OA cartilage [14, 16, 30]. Again, there is inconsistency among studies concerning the areas of IGF1R expression within OA cartilage. Such inconsistencies might stem from differences in the evaluation methods, as these previous studies were all histological studies involving relatively small numbers of samples. In the present study, we evaluated the IGF1R expression in OA and control cartilage by a more quantitative method using a reasonable number of samples, and clearly demonstrated that the expression of IGF1R was significantly reduced in OA cartilage. The reduction in expression was more notable in degenerated areas than in preserved areas, which was confirmed by the result of a protein analysis.

A similar trend was observed for IRS1. Although the expression of IRS1 did not change markedly in preserved areas of OA cartilage compared to the control, its expression was substantially reduced in degenerated areas. This regional difference within OA cartilage was, again, confirmed by a protein analysis. Upon engagement with IGFs, IGF1R phosphorylates IRS1, which then activates the PI3K/AKT/mTOR pathway to promote cartilage matrix synthesis [26, 27]. Therefore, a reduction in the expression of IGF1R as well as IRS1 in degenerated areas of OA cartilage implies that the IGF signaling may be highly attenuated in these areas.

In 1994, Dore et al. first reported that the chondrocytes from OA cartilage respond poorly to IGF-1 due to an increased IGFBP expression [16]. Since then, many researchers have investigated the expression of IGFBPs in OA cartilage, and an enhanced expression of IGFBP-2, IGFBP-3, IGFBP-4, and IGFBP-5 in human OA cartilage has been reported [18, 30, 33]. In the present study, we evaluated the expression of six IGFBP genes in OA and control cartilage, and found that expression of IGFBP-1, IGFBP-2, and IGFBP-3 was enhanced in OA cartilage compared to the control cartilage, while that of IGFBP-4, IGFBP-5, and IGFBP-6 was not. Furthermore, in our analysis, an interesting trend was noted where the expression of all six IGFBP genes tended to be higher in degenerated areas than in preserved areas within OA cartilage. The analysis of urea extracts confirmed this trend for IGFBP-1, IGFBP-3, and IGFBP-6. These results may suggest the presence of a common regulatory mechanism underlying IGFBP expression within OA cartilage. Since IGFBPs are critical components of the IGF system, the identification of such a mechanism may be a goal worth pursuing.

Given that all of the above results indicated attenuated IGF signaling in degenerated areas of OA cartilage, we next conducted an explant culture experiment to examine that possibility. We found that matrix gene expression in the explants from preserved areas was considerably reduced by the inhibition of AKT signaling, while such reduction was not apparent with the explants from degenerated areas. As mentioned earlier, AKT signaling is responsible for the promotion of chondrocyte matrix synthesis by IGFs [26, 27]. Therefore, this result was understood to support the notion that the decline in matrix gene expression in degenerated areas is caused by the attenuation of IGF signaling. In this experiment, we also noticed that in the explants from preserved areas, the suppression of type II procollagen expression by the inhibitor was greater than that of aggrecan expression, suggesting that type II procollagen expression is more dependent on IGF signaling than aggrecan expression. A previous study reported that, in articular chondrocytes, type II procollagen is a gene more responsive to IGF-1 than aggrecan [9]. The discrepancy seen in the suppression of expression by the inhibitor might be due to this difference in response to IGF between those genes.

Since IGF1R plays pivotal roles in various biological events, the regulatory mechanisms concerning its expression have been the focus of intense research. Thus far, several transcription factors have been found to regulate the IGF1R expression [28, 34]. Among them, Sp1 is known to be a critical regulator of the IGF1R expression [28, 29]. Based on that knowledge, we performed a gene expression analysis and chondrocyte culture experiments, and obtained results indicating that the expression of IGF1R was regulated by Sp1 in OA cartilage. Interestingly, the results also indicated that the expression of IRS1 is regulated by Sp1. Although relatively little is known about the regulation of the IRS1 expression, again, the involvement of Sp1 is suggested [35]. We consider it very reasonable that the IRS1 expression is regulated together with that of IGF1R in OA cartilage because these molecules are closely linked in the IGF system.

Taken together, our findings suggested that at least three changes in the IGF system, namely, a reduced IGF expression, increased IGFBP expression, and reduced IGF1R and IRS1 expression might underlie the reduced cartilage matrix synthesis in degenerated areas of OA cartilage. Of course, changes in chondrocyte metabolism within OA cartilage are likely a very complex event and cannot be explained simply by the modulation of the IGF system. Nonetheless, we believe that our current findings will help those who are engaged in the study of this common, but tenacious disease.

Taken together, our findings suggested that at least three changes in the IGF system, namely, a reduced IGF expression, increased IGFBP expression, and reduced IGF1R and IRS1 expression might underlie the reduced cartilage matrix synthesis in degenerated areas of OA cartilage. Although these results seem convincing, this study has several limitations as well. First, though enhanced IGFBP expression in degenerated areas has been shown, to what extent IGF signaling is inhibited by each of the proteins remains unclear. Again, although the suppression of SP1 expression was shown to be responsible for the reduced IGF1R and IRS1 expression in degenerated areas, whether or not their diminished expression can be solely ascribed to the decline in SP1 expression has not been clarified. However, despite these and other possible limitations, we believe that our current findings will help those who are engaged in the study of this common, but tenacious disease.

## Conclusions

To determine the mechanisms underlying the reduced matrix synthesis in degenerated areas of OA cartilage, we performed gene expression and protein analyses of human cartilage samples, and revealed that the expression of IGF-1, IGF-2, IGF1R and IRS1 was suppressed, while that of six IGFBP genes was elevated in degenerated areas of OA cartilage compared to macroscopically intact areas. This result suggests that the reduction in cartilage matrix synthesis in degenerated areas may be ascribed, at least in part, to attenuated IGF signaling in these areas. We further investigated the mechanisms underlying the reduced IGF1R and IRS1 expression in degenerated areas, and found that their expression was regulated by Sp1 in OA cartilage and that a decline in the Sp1 expression might be responsible for the reduction in the IGF1R and IRS1 expression in those areas. These findings may be worth noting when exploring therapies to inhibit the loss of cartilage matrix in OA.

## Data Availability

The datasets obtained and analyzed during the current study are available from the corresponding author on reasonable request.

## Abbreviations

LCM: Laser capture microdissection
OA: Osteoarthritis
qPCR: Quantitative polymerase chain reaction
